# Contrasting effects of fungicide and herbicide active ingredients and their formulations on bumblebee learning and behaviour

**DOI:** 10.1242/jeb.245180

**Published:** 2023-03-14

**Authors:** Linzi J. Thompson, Jane C. Stout, Dara A. Stanley

**Affiliations:** ^1^ School of Agriculture and Food Science, University College Dublin, Dublin 4, Ireland; ^2^ Earth Institute, University College Dublin, Dublin 4, Ireland; ^3^ School of Natural Sciences, Trinity College Dublin, Dublin 2, Ireland

**Keywords:** Glyphosate, Bee, Pesticide, Prothioconazole, Roundup, Proline, *Bombus terrestris audax*, Co-formulant

## Abstract

Fungicides and herbicides are two of the most heavily applied pesticide classes in the world, but receive little research attention with regards to their potential impacts on bees. As they are not designed to target insects, the mechanisms behind potential impacts of these pesticides are unclear. It is therefore important to understand their influence at a range of levels, including sublethal impacts on behaviours such as learning. We used the proboscis extension reflex (PER) paradigm to assess how the herbicide glyphosate and the fungicide prothioconazole affect bumblebee olfactory learning. We also assessed responsiveness, and compared the impacts of these active ingredients and their respective commercial formulations (Roundup Biactive and Proline). We found that learning was not impaired by either formulation but, of the bees that displayed evidence of learning, exposure to prothioconazole active ingredient increased learning level in some situations, while exposure to glyphosate active ingredient resulted in bumblebees being less likely to respond to antennal stimulation with sucrose. Our data suggest that fungicides and herbicides may not negatively impact olfactory learning ability when bumblebees are exposed orally to field-realistic doses in a lab setting, but that glyphosate has the potential to cause changes in responsiveness in bees. As we found impacts of active ingredients and not commercial formulations, this suggests that co-formulants may modify impacts of active ingredients in the products tested on olfactory learning without being toxic themselves. More research is needed to understand the mechanisms behind potential impacts of fungicides and herbicides on bees, and to evaluate the implications of behavioural changes caused by glyphosate and prothioconazole for bumblebee fitness.

## Teaser

**Summary:** The fungicide prothioconazole and herbicide glyphosate at field-realistic doses may not negatively impact olfactory learning ability of bumblebees in a lab setting, but glyphosate may cause changes in bumblebee responsiveness.

## INTRODUCTION

Pollinators, including bees, are important for maintaining biodiversity and food security at a global level (Klein et al., 2007). Bees are under threat from a variety of stressors, one of which is the use of pesticides (Potts et al., 2010). Insecticides are a class of pesticides designed to kill insect pests and as such are widely studied with regards to their toxicity and sub-lethal effects on non-target insects, especially bees, with a particular focus on certain classes such as the neonicotinoids (Lundin et al., 2015). However, globally, fungicides and herbicides are more heavily used than insecticides (in terms of kg applied). Just as with insecticides, bees may come into contact with plants recently treated with these pesticides and as such consume contaminated floral resources (Gradish et al., 2019; Zioga et al., 2020, 2022; Thompson et al., 2022). Despite this, our knowledge of whether the use of fungicides and herbicides has impacts for bees is lacking (Cullen et al., 2019; FAOSTAT, 2020), most likely as these classes are not designed to target insect pests. However, increasingly, evidence is suggesting that there may be behavioural and physiological implications of exposure to certain substances within these pesticide classes on bees (Cullen et al., 2019), and so it is important to understand any impacts to guide pesticide risk assessment and bee conservation.

When pesticides are applied, it is usually as part of a formulation. Co-formulants added to pesticide formulations aid in the delivery and efficacy of the pesticide active ingredient. However, most research on bees and pesticides focuses on active ingredients only, and little is known about how these impacts are modulated by co-formulants or whether there are impacts of co-formulants themselves. Some initial evidence suggests that these co-formulants may also affect bees (Straw et al., 2022) as they can have the ability to cause changes in microbiota or changes at the physiological level (e.g. Straw and Brown, 2021; Cullen et al., 2023). However, we have very little understanding of the mechanisms behind this, and so testing the impacts of both formulations and active ingredients is important because it may aid understanding of the role of co-formulants in pesticidal effects.

Learning ability is key to bumblebee behaviour and their ultimate survival, as it allows bees to forage efficiently (Raine and Chittka, 2008) and is related to the length of their foraging career (Evans et al., 2021), amongst other tasks. If bumblebee learning is impaired after pesticide exposure, it is possible it may affect their ability to gather food resources for their colony or even return to their colony, with implications for colony fitness. Some insecticides have been found to have effects on bumblebee learning, such as the neonicotinoid thiamethoxam (Stanley et al., 2015a) and the sulfoximine sulfoxaflor (Siviter et al., 2019; Vaughan et al., 2022). The known mechanism for these insecticides is action on the insect nervous system, including on the acetylcholine receptors in the bee brain, which are associated with learning and memory, and so impacts on these behaviours are not surprising. Related to learning ability is a bee’s ability to respond to sucrose or its motivation to respond. If bees do not respond to sucrose, this could indicate sensory or motivational impairments, and changes in responsiveness have been observed as a result of exposure to other pesticides (e.g. Frost et al., 2013; Demares et al., 2018; Lämsä et al., 2018; Muth and Leonard, 2019). Changes in responsiveness may have consequences for colony fitness, where bees may not collect sufficient food either because they are not being stimulated to forage by flowers or because they simply lack the motivation to collect from them. Therefore, responsiveness, in addition to learning, may be important to explore in the context of fungicide and herbicide use.

As the modes of action of fungicides and herbicides in bees are not well understood, we therefore have little understanding of the potential hazards. It is important to investigate impacts of these substances on a range of behaviours, including learning, memory and responsiveness, as this could guide us in understanding which pathways, or areas of bee anatomy, are being affected. Additionally, some pesticides such as clothianidin were shown to negatively affect learning in honeybees but not bumblebees (Piiroinen and Goulson, 2016), suggesting species-specific effects. So, whilst negative effects of glyphosate on honeybee learning have been observed (Herbert et al., 2014; Mengoni Goñalons and Farina, 2018), we might not expect to see the same effect in bumblebees and this requires further research.

Here, we investigated how bumblebee learning and responsiveness is affected by chronic exposure to field-relevant levels of a herbicide (glyphosate) and a fungicide (prothioconazole), as both an active ingredient and in formulation, when colonies are exposed. Responsiveness to sucrose was assessed alongside learning ability using the proboscis extension reflex (PER) paradigm in three different experiments. PER is used to assess the olfactory learning performance of bees, and although it is commonly used to evaluate olfactory learning in honeybees (e.g. Williamson and Wright, 2013; Wright et al., 2015), it is also becoming a popular tool for use in bumblebees (Riveros and Gronenberg, 2009; Siviter et al., 2018).

Within a bumblebee colony, many complex interactions occur and, as these insects are eusocial, it is important to study how exposure to pesticides may affect not only individuals directly but also individuals from an exposed colony. We therefore used both microcolony- and colony-level exposure in our work. This is of particular importance because knowledge of how fungicides and herbicides affect bumblebees when exposed at the colony level is particularly lacking (Cullen et al., 2019).

## MATERIALS AND METHODS

### Compound choice

We chose to investigate the impacts of the herbicide glyphosate and the fungicide prothioconazole on bees, as the most widely used herbicide and fungicide compounds in Ireland (López-Ballesteros, et al., 2022). Glyphosate is also the most applied herbicide in the world (in terms of kg used; Benbrook, 2016), and is a broad-spectrum and non-selective herbicide employed for a variety of uses in agriculture, but also heavily used in domestic and amenity settings (Duke and Powles, 2008). Bees can also come into contact with glyphosate in the environment; for example, bees forage on plants treated with glyphosate and return nectar and pollen containing residues to their colony (Thompson et al., 2014; 2022). Prothioconazole is a broad-spectrum fungicide, commonly applied to a variety of crops such as oilseed rape and cereals (FAO, 2021), and is widely used across the EU (López-Ballesteros et al., 2022). Residues of prothioconazole have also been measured in nectar and pollen collected by bees (see below). We chose to compare the effects of the active ingredients with those of the commercial glyphosate formulation Roundup Biactive and the prothioconazole formulation Proline, as both products contain only one pesticidal active ingredient (glyphosate or prothioconazole). Proline is widely available for agricultural use, and Roundup Biactive is available for non-professional consumers. Currently, there is evidence to suggest that honeybee learning can be impaired as a result of exposure to glyphosate (Mengoni Goñalons and Farina, 2018), although there is no research exploring whether this herbicide affects learning in bumblebees. Prothioconazole lacks research attention with regards to impacts on bees in general (Cullen et al., 2019).

### Bumblebee learning

To test the effects of fungicides and herbicides on bumblebee learning, three experiments were carried out (Table 1) on *Bombus terrestris audax* (Harris 1776). No ethical approval was required to work with bees.

**Table 1.**
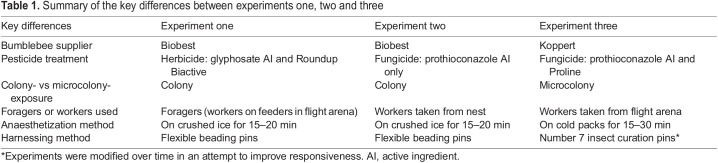
Summary of the key differences between experiments one, two and three

### Experiment one: colony-level herbicide

#### Bumblebees

Biobest (Westerlo, Belgium) *B. terrestris audax* research colonies (*n*=24) were obtained in July 2021 and maintained on their original feeder until 48 h prior to pesticide treatment. At this point, colonies were provided with 30% w/v sucrose solution, until given their treatment solution. Colonies also received unspiked pollen bread consisting of ground pollen pellets and 50% sucrose solution every 2–3 days. Colonies received pesticide spiked/control treatment between 1 and 3 weeks after arrival in the lab. Colonies were given access to flight arenas for 1 day on arrival in the lab before receiving pesticide treatment, and a further 2, 3 or 4 days during pesticide treatment (on days 2/3/4 out of the 5 days of pesticide exposure). It is likely that selected bees had fed on a chronic dose of contaminated sucrose as colonies were exposed for 3–4 days, and foragers were selected from a spiked feeder and as such they had at least one acute dose.

#### Pesticide treatment

Bumblebees received one of four treatments: sucrose control, or sucrose spiked with 1 mg kg^−1^ glyphosate active ingredient (glyphosate AI: CAS: 1071-83-6, >95% purity; Molekula, Darlington, UK), 1 mg kg^−1^ of glyphosate active ingredient in Roundup Biactive (Roundup low) or 21,600 mg kg^−1^ of glyphosate active ingredient in Roundup Biactive (Roundup high). Only sucrose solution was spiked with pesticide – pollen remained unspiked. Colonies received their treatment solution for 3–4 days before bees were selected for PER trials, to mimic a situation where bees would feed on a treated food source for this length of time. Glyphosate has been recorded at 1–1.30 mg kg^−1^ in nectar stores of honeybee colonies, 2.78–31.3 mg kg^−1^ in the honeybee stomach, and 87.2–629 mg kg^−1^ in pollen after honeybees foraged on treated plants over a 7 day period (Thompson et al., 2014). Additionally, glyphosate has been detected in corbicular pollen at 5 mg kg^−1^ up to 3 days post-spray (Thompson et al., 2022). Therefore, we chose a 1 mg kg^−1^ concentration for both active ingredient and formulation treatments to mimic conservative but environmentally relevant concentrations in nectar that bees could feed on (see Table S3). The Roundup high concentration (21,600 mg kg^−1^) was chosen to reflect the concentration of glyphosate in Roundup Biactive spray as applied in the field based on the recommended spray rate from the label. This therefore reflected a ‘worst-case’ scenario, where bees would consume spray through nectar directly with little or no dilution or degradation. It was not possible to compare this Roundup high treatment with active ingredient only at the same concentration, as glyphosate is insoluble at 21,600 mg kg^−1^ without further co-formulants.

Pesticide or control solutions were contained in gravity feeders which the entire colony had access to. During the exposure period, colonies also had access to flight arenas that contained feeders with only their treatment for the final 2 days of the exposure period only. One flight arena was used per treatment for the whole experiment to prevent cross-contamination. Foraging bees were captured from feeders in the flight arenas and harnessed immediately for PER trials, on the final day of exposure (day 3 or 4).

### Experiment two: colony-level fungicide

#### Bumblebees

Biobest *B. terrestris audax* research colonies (*n*=10) were obtained in July 2021 and maintained on their original feeder until 48 h prior to pesticide treatment, where they received 30% w/v sucrose solution and unspiked pollen in the form of pollen bread (same as in experiment one). Colonies received pesticide treatment approximately 1 month after arrival in the lab. Colonies received their treatment solution for 3–4 days, before being used in PER trials. As colonies were exclusively fed their treatment for 3–4 days, it is likely that all bees received some form of pesticide contamination.

#### Pesticide treatment

Information on prothioconazole in the environment is lacking (Zioga et al., 2020), but it has been recorded at a range of 10–356 µg kg^−1^ in pollen (Roszko et al., 2016; Bokšová et al., 2021) and 0.69–0.009 mg kg^−1^ in nectar over a 7 day period following application of Proline (Wallner, 2009). Based on this, we chose to expose bees to 300 µg kg^−1^ (see Table S3), which represents the upper levels of this field-relevant range. As this pesticide is not well studied with regards to its impact on bees (Cullen et al., 2019), it was important to first find out whether these higher field-relevant concentrations have an effect.

Prothioconazole requires a solvent for dissolving, and therefore an equal amount of acetone was used in both control and pesticide treatment solutions. This resulted in the following two treatments: sucrose solution spiked with 0.3% acetone control and sucrose solution spiked with 300 µg kg^−1^ prothioconazole active ingredient dissolved in 0.3% acetone (CAS: 178928-70-6, >95% purity; Biosynth Carbosynth, Compton, UK). Both treatments were exclusively given to bees through gravity feeders which colonies had access to for the entire 3–4 day treatment period. The concentration of acetone was kept to below 5% (v/v), as per OECD (2017) guidelines. Only sucrose solution was spiked with pesticide – pollen remained unspiked. Bumblebees were then randomly selected from the nest and harnessed. Because the bumblebees showed little or no activity when given access to a flight arena, it was not possible to select known foragers.

### Experiment three: microcolony-level fungicide

#### Microcolonies

Forming a microcolony entails removing a small number of workers from a colony and putting them together in a small box where one worker will eventually become dominant and lay eggs. Microcolonies are commonly employed for assessing pesticide effects on bees (e.g. Piiroinen and Goulson, 2016; Vaughan et al., 2022), allowing the same colony to be used across pesticide treatments, reducing the impact of inter-colony variation. Koppert (Unichem, Dublin, Ireland) *B. terrestris audax* colonies (*n*=5) were obtained in April 2021 as Biobest ones were unavailable. On arrival, they were maintained on the original feeder and fed pollen bread every 2–3 days, as in experiments one and two. Colonies were given access to a flight arena where workers were randomly selected and put into queenless microcolonies (for a maximum of 6 days) within 1–2 weeks of arrival. Bees were not given any nest material and as such did not have any nectar stores to feed from. As bees were kept in the microcolony for several days, they had to feed on the contaminated sucrose solution.

#### Pesticide exposure

This experiment contained five treatments: sucrose control, acetone control (as in experiment two), Proline containing 300 µg kg^−1^ prothioconazole active ingredient (Proline low), Proline containing 1750 mg kg^−1^ prothioconazole active ingredient (Proline high), and 300 µg kg^−1^ prothioconazole active ingredient (prothioconazole AI: CAS: 178928-70-6, >95% purity; Biosynth Carbosynth). As in experiment two, prothioconazole was dissolved in acetone and therefore an acetone control containing the same concentration of acetone (0.3%) was used, again below 5% (v/v) as per OECD (2017) guidelines. The sucrose control was used to allow comparison with commercial formulation Proline. Prothioconazole concentrations were selected based on the justification in experiment two and were compared with the same concentration of the formulation Proline (Proline low). As with glyphosate in experiment one, we also wanted to investigate any impacts of the recommended spray rate of Proline as a ‘worst case’ exposure, resulting in the Proline high treatment (1750 mg kg^−1^) based on the label-recommended application rate. However, again because of solubility issues, we could only test the spray rate concentration when in formulation. Only sucrose solution was spiked with pesticide, while pollen remained unspiked.

One microcolony (containing 10–15 workers) per treatment was made from each colony (*n*=40 total), meaning each natal colony was equally distributed across treatments. Microcolonies were fed their treatment solution for 3–4 days before being harnessed and used in PER trials. However, not all microcolonies produced harnessed bees that were responsive for the PER trials, resulting in bees from a total of 37 microcolonies in the final analysis.

### Bumblebee conditioning and harnessing

#### Harnessing

To harness bees across all experiments, bumblebees were contained in individual vials and placed on cold packs for 15–30 min (experiment three only) or ice for 15–20 min (experiments one and two) until immobile. Bees were then harnessed using a modified syringe with a V-shaped notch cut out of the front, with damp cotton wool in the bottom, as in Stanley et al. (2015a). Bees were held in place by pins placed between the thorax and the head and secured by duct tape. In experiment three, thicker, non-flexible pins were used but these were switched for finer and more flexible pins in experiments one and two (see Fig. S1), which may have contributed to bees having a higher responsiveness in those experiments (Table 1). Once bees were harnessed, they were fed until satiation with 40% w/v sucrose solution and left overnight in a dark room. The following morning, responsiveness of bees was tested immediately prior to the PER trials, and only bees that extended their proboscis in response to antennal stimulation with 50% sucrose solution (i.e. were responsive) were used; the recorder was blind to the treatment of bees.

#### Learning ability

The PER paradigm was used to assess the learning ability of bumblebees in all three experiments, following methods in Stanley et al. (2015a). In olfactory PER tests, bumblebees are conditioned to associate an odour with a reward (50% w/v sucrose solution). Bees were conditioned individually, inside an odour extraction hood, to ensure the odour was removed immediately after presentation. A glass odour tube containing 1 µl of lavender essential oil was placed 3 cm away from and pointed towards the bee. Lavender oil was chosen because of its usage in other bumblebee assays (e.g. Lawson et al., 2017; Telles et al., 2017), and because one of the main volatile organic compounds in lavender is linalool (Nematollahi et al., 2018), which is commonly used in PER and other learning assays (e.g. Sommerlandt et al., 2014; Piiroinen and Goulson, 2016; Muth et al., 2019). The odour tube was changed every 20–30 uses to ensure a consistent odour. Flow rate, volume of air and duration of odour presentation were controlled using a programmable logic controller.

Once an individual bee was placed in the odour extraction hood, the bee was left to acclimatise for 5 s and was then presented with odour for 10 s. At 6 s into the odour presentation, a 0.8 µl droplet of 50% sucrose solution was touched against both antennae rapidly, using a Gilmont syringe, and if the bee extended its proboscis, it was allowed to consume the entire droplet. Bees were presented with the odour 15 times with an inter-trial interval of 12 min. Once the bee was successfully conditioned to the odour (i.e. had ‘learnt’ the association), it would extend its proboscis when the odour was presented in anticipation of the reward being offered. During each presentation, we recorded whether the bee had learnt the association, extended its proboscis in response to antennal stimulation (i.e. did not learn but responded to the sucrose stimulus) or did not respond (i.e. the bee did not show either a learnt response or a response to antennal stimulation) (see Supplementary Materials and Methods). In total, there were six runs of PER tests for experiment one, three for experiment two and eight for experiment three. During experiments one and two, bees were from multiple natal colonies; in experiment three, all bees were from the same colony. Each run consisted of 15 odour presentations (trials). The intertegular distance of all bees was measured as a proxy for body size (Hagen and Dupont, 2013), following completion of PER tests. A diagram of the PER set up can be found in Fig. S1.

### Data analysis

A variety of parameters were recorded to determine how learning performance was affected during the PER experiments. Using methods modified from Stanley et al. (2015a), we assessed the trainability of bees (their ability to learn the association or not – binary), learning level (the number of presentations where bees demonstrated a learnt response, which included only bees which learnt the association) and learning rate (the number of responses to odour presentations until bees learnt the association, which included only bees which learnt the association). We also evaluated responsiveness, determined as the number of times bees failed to extend their proboscis once stimulated with sucrose solution through the PER experiment, in two ways: the number of non-responses each bee had, and the number of bees which had a non-response (binary).

All bees that did not respond (i.e. did not extend their proboscis and feed when their antennae were stimulated) to a minimum of five odour presentations during the PER trials were considered non-responsive and removed from analysis of trainability, learning rate and learning level. This resulted in 31 bees being removed from experiment one (sucrose control *n*=5 bees, glyphosate AI *n*=10 bees, Roundup low *n*=7 bees and Roundup high *n*=9 bees), two bees being removed from experiment two (control and prothioconazole) and 27 being removed from experiment three (sucrose *n*=3, acetone *n*=4, prothioconazole AI *n*=9, Proline low *n*=1 and Proline high *n*=10). All bees were included in analysis of responsiveness. All data were summarised so there was one measurement per bee.

Models were used to test relationships between the parameters recorded as response variables, and treatment and body size (intertegular distance) and their interaction as explanatory variables, in each experiment. Body size in bumblebees is highly variable and was considered a co-variate as some research suggests it can influence learning ability (Ings et al., 2005; Worden et al., 2005; Stanley et al., 2015a). Poisson models were used for count data and negative binomial models (distribution: nbinom1 or nbinom2) were used to control for overdispersion, depending on best model fit. Models using count data were validated by viewing *Q*–*Q* plots and residuals versus fitted plots. Binomial models were validated using DHARMa (https://CRAN.R-project.org/package=DHARMa). Model simplification was carried out by removing the interaction between body size and treatment, and body size, if not significant (*P*>0.05), using likelihood ratio tests, until there was only treatment left, or both variables or the interaction was significant (Colegrave and Ruxton, 2017). Model summaries for simplified models and 95% confidence intervals are reported in Table S1. The estimate (presented in Table S2) was used to further interpret the magnitude of effect as well as the percentage change in raw means, using the control as the base value. All graphs were plotted using ggpubr, patchwork and ggrepel (https://CRAN.R-project.org/package=ggpubr; https://CRAN.R-project.org/package=patchwork; https://CRAN.R-project.org/package=ggrepel).

#### Experiments one and two (herbicide and fungicide colony level)

In experiment one, 100 bees entered PER trials with the following final sample sizes remaining for data analysis: sucrose control *n*=23, glyphosate AI *n*=13, Roundup low *n*=20 and Roundup high *n*=13. In experiment two, 65 bees entered PER trials, with *n*=33 and 30 remaining in the analysis of prothioconazole and acetone control treatments, respectively. Generalised linear mixed effects models from the package glmmTMB (Brooks et al., 2017), using the function glmmTMB, were used to analyse all data. Run nested in colony was specified as a random intercept. The same explanatory variables were used and simplified and validated as above. In experiment two, only two bees that received the glyphosate AI treatment learnt the association and therefore the sample was not sufficient for analysis of learning level and rate. Linear mixed effects models using the lme function from the nlme package (https://CRAN.R-project.org/package=nlme) were used to assess body size across treatments, with the same random effects structure as above.

#### Experiment three (fungicide microcolony)

Of the 105 bees that entered PER trials, the following remained for data analysis: sucrose control *n*=18, acetone control *n*=21, prothioconazole AI *n*=26, Proline low *n*=22 and Proline high *n*=17. One additional bee from the acetone control treatment was excluded because of technical issues with data recording (final, *n*=20). Models were run as in experiments one and two; however, the random effects structure was different because of differences in experimental design. Microcolony nested in run nested in natal colony was specified as a random intercept. Linear mixed effects models using the lme function from the nlme package were used to assess body size across treatments, with the same random effects structure as above.

## RESULTS

Overall, there was little effect of pesticide treatment, including active ingredients and commercial formulations, on the olfactory learning ability of bumblebees. However, in experiment one, exposure to the active ingredient glyphosate did reduce the responsiveness of bees. In experiment two, bees exposed to prothioconazole that learnt the association between odour and reward had more learnt associations than control bees, although this same pattern with prothioconazole was not evident in experiment three.

### Experiment one: colony-level herbicide

**Fig. 1.**
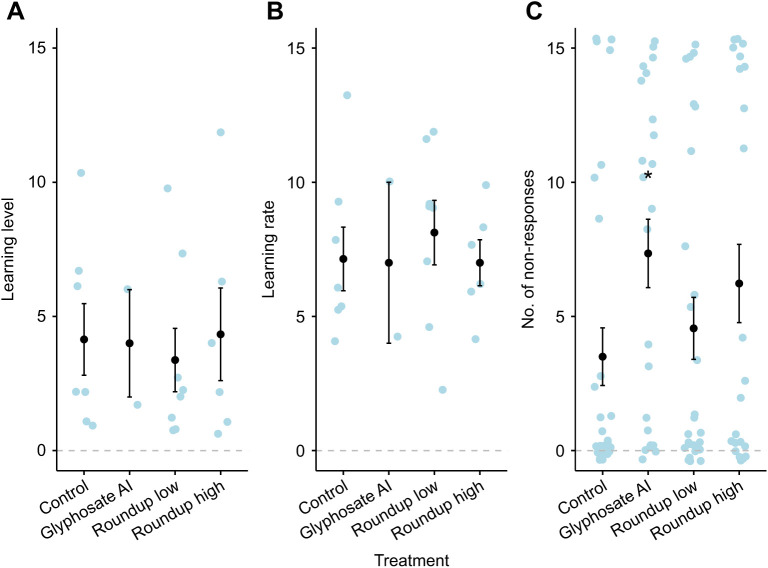
**Responses to glyphosate trials (experiment one).** (A) Learning level (the number of learnt responses); (B) learning rate (the number of odour presentations until bees first learnt); and (C) the number of non-responses to antennal stimulation. Bumblebees were exposed to glyphosate active ingredient (glyphosate AI), the commercial formulation Roundup Biactive at nectar residue and spray rate concentrations (Roundup low and high, respectively), or control solution (sucrose) at the colony level before olfactory testing using the proboscis extension reflex (PER) was carried out. Exposure to glyphosate AI significantly reduced the responsiveness of bumblebees (*P=0.0002), but as this resulted in small sample sizes, this treatment could not be included in the analysis of learning level and learning rate. Black circles and bars are means±s.e.m.; blue circles are raw data (individual bees). The grey dashed line depicts the zero response.

In experiment one, the number of non-responses was affected by both treatment (glmmTMB: χ^2^_3_=8.21, *P*=0.042; Fig. 1) and body size (glmmTMB: χ^2^_1_=13.80, *P*=0.0002), separately and not interacting, where glyphosate-treated bees on average had 110% more non-responses compared with controls (Tukey: *t*-ratio=−2.95, *P*=0.021; Table S2) and smaller bees were more likely to show non-responses. The proportion of bees that had at least one non-response was also significantly affected by both treatment (glmmTMB: χ^2^_3_=8.21, *P*=0.042) and bee size (glmmTMB: χ^2^_1_=11.91, *P*=0.0006), separately and not interacting: 117% more bees had at least one non-response from glyphosate-treated colonies compared with controls, with an estimated 2.12 times higher likelihood of at least one non-response compared with control treated bees (Tukey: *t*-ratio=−2.65, *P*=0.046; Table S2), and larger bees had fewer non-responses. There was no difference in either non-response variable between Roundup treatments and control. Learning level of bees was not affected by Roundup or glyphosate (glmmTMB: χ^2^_2_=2.13, *P*= 0.35), but body size did have a marginally significant effect (glmmTMB: χ^2^_1_=4, *P*=0.046), where smaller bees from all three treatments were more likely to have a learnt response. There was also no significant effect of either Roundup treatment on the learning rate of bees (glmmTMB: χ^2^_2_=4.58, *P*=0.10); however, there was a significant effect of body size, where smaller bees learnt faster (glmmTMB: χ^2^_1_=8.26, *P*=0.004).

Trainability of bees was unaffected by glyphosate AI and Roundup treatment (glmmTMB: *F*_3_=3.47, *P*=0.32). Following the drop in responsiveness in the glyphosate treatment, sample size of bees from this treatment was too low to evaluate impacts on learning level and rate. Body size did not differ across treatments (lme: χ^2^_4_=5.75, *P*=0.13).

### Experiment two: colony-level fungicide

**Fig. 2.**
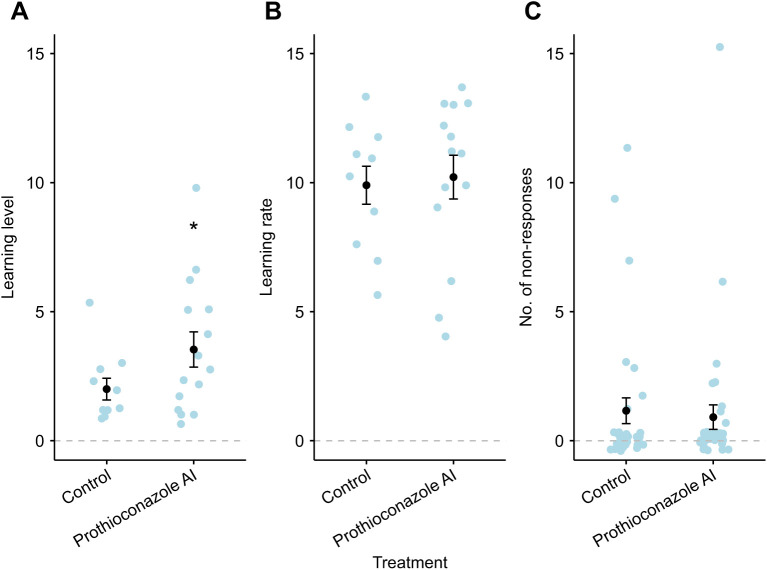
**Responses to prothioconazole trials (experiment two).** (A) Learning level (the number of learnt responses); (B) learning rate (the number of odour presentations until bees first learnt); and (C) the number of non-responses to antennal stimulation. Bumblebees were exposed to either prothioconazole active ingredient (prothioconazole AI) or control solutions, at a field­-relevant concentration, at the colony level before olfactory testing using the PER. The increase in learning level for prothioconazole AI in A was significant (*P=0.026). Black circles and bars are means±s.e.m.; blue circles are raw data (individual bees). The grey dashed line depicts the zero response.

**Fig. 3.**
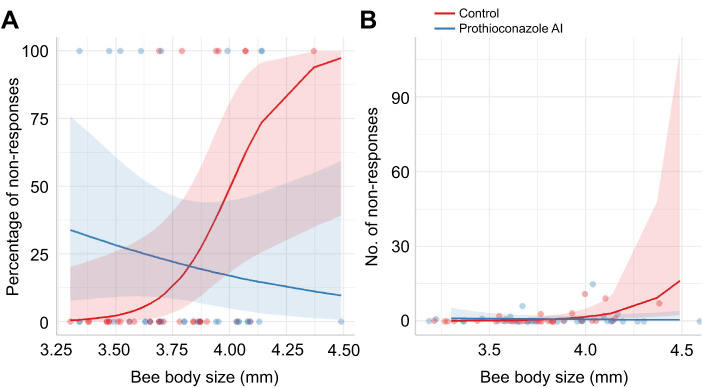
**Model predictions where interactions between body size and fungicide treatment were found (experiment two).** (A) The percentage of non-responses during experiment two against body size, showing that the control and prothioconazole AI treatment caused opposite effects in the responsiveness of bees (*P*=0.016). (B) The number of non-responses in experiment two against body size. Larger bees from the control treatment had more non-responses than those exposed to prothioconazole AI (*P*=0.022). In both graphs, the red and blue shaded areas are the 95% confidence intervals for each treatment.

The learning level of bees differed as a result of prothioconazole AI treatment (glmmTMB: χ^2^_1_=4.98, *P*=0.026) and body size (glmmTMB: χ^2^_1_=4.34, *P*=0.037), but there was no interaction between them. Among the bees that learnt, prothioconazole-treated bees had a 76.5% higher level of learning compared with controls (Tukey: estimate 0.689, *t*-ratio=−2.452, *P*=0.024) and there was a trend for smaller bees to have more learnt responses. However, one bee performed much better than the rest and after removal of this data point the model conclusions changed and this was no longer significant (glmmTMB: χ^2^_1_=1.38, *P*=0.24). Despite this, there was still a trend where prothioconazole-treated bees had a 53.5% higher level of learning. Neither prothioconazole nor body size had an effect on trainability (glmmTMB: χ^2^_1_=10.97, *P*=0.32) or learning rate (glmmTMB: χ^2^_1_=0.06, *P*=0.81; Fig. 2) of bees. However, there was a significant interaction between body size and treatment when analysing the number of non-responses (glmmTMB: χ^2^_2_=7.60, *P*=0.022; Fig. 3), where larger bees from the control treatment were more likely to have a non-response – but this was not reflected in the prothioconazole treatment. Similarly, there was a significant interaction between treatment and body size in the proportion of bees that had a non-response (glmmTMB: χ^2^_3_=10.28, *P*=0.016), where larger control treated bees were more likely to have a non-response, but the reverse was true in the prothioconazole treatment. Body size did not differ across treatments (lme: χ^2^_4_=1.28, *P*=0.26).

### Experiment three: microcolony-level fungicide

**Fig. 4.**
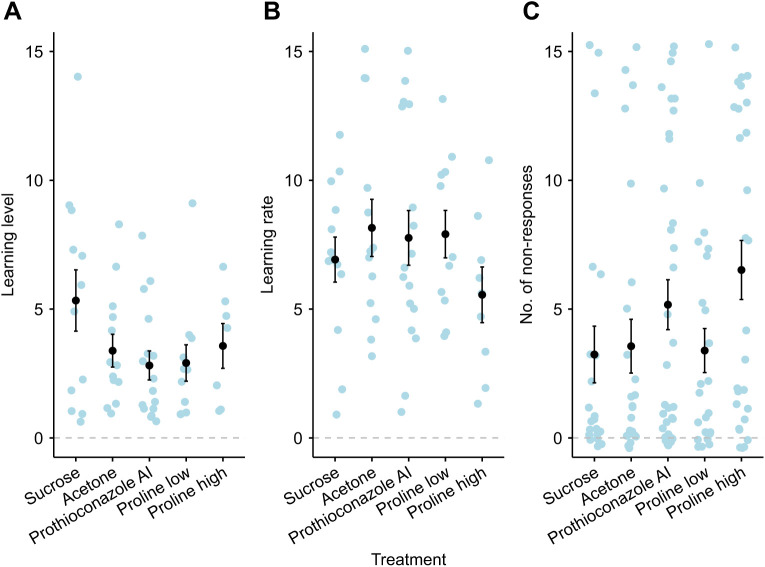
**Response to prothioconazole and Proline (experiment three).** (A) Learning level (the number of learnt responses); (B) learning rate (the number of odour presentations until bees first learnt); and (C) the number of non-responses to antennal stimulation. Bumblebees were exposed to prothioconazole AI, the commercial formulation Proline at filed-relevant and spray rate concentrations (Proline low and high, respectively) or control solutions (sucrose and 0.3% acetone), at the microcolony level. No significant differences were found between any of the comparisons made. Black circles and bars are means±s.e.m.; blue circles are raw data (individual bees). The grey dashed line depicts the zero response.

Neither the fungicidal active ingredient prothioconazole nor the commercial formulation Proline, or body size, had an effect on bee responsiveness (number of responsive bees: glmmTMB: χ^2^_1_=0.87, *P*=0.35; proportion of bees which showed at least one non-response: glmmTMB: χ^2^_4_=3.68, *P*=0.45), trainability (glmmTMB: χ^2^_4_=2.73, *P*=0.61) or learning rate (glmmTMB: χ^2^_4_=0.94, *P*=0.92; Fig. 4). Learning level was not affected by pesticide treatment, but body size did have an effect (glmmTMB: χ^2^_1_=4.79, *P*=0.029), where bigger bees had a higher number of learnt responses, across all treatments. Body size did not differ across treatments (lme: χ^2^_5_=2.13, *P*=0.71).

## DISCUSSION

Learning and memory are key behaviours essential to how bumblebees function, which have been shown to be impacted by certain insecticides. Here, we found no evidence of olfactory learning impairment by the tested fungicide or herbicide active ingredients or formulations at both field-relevant and spray rate concentrations, but we did find that learning level could be enhanced by prothioconazole in some conditions, and responsiveness could be reduced by glyphosate.

As glyphosate is the most heavily used herbicide in the world, research on its potential impacts on bees is increasing (Cullen et al., 2019). We observed that glyphosate AI at a field-relevant concentration reduced the responsiveness of bumblebees to antennal stimulation. A reduction in responsiveness could be a result of many different mechanisms. Glyphosate, in addition to other pesticides such as thiamethoxam, has previously been found to reduce the sucrose responsiveness of honeybees (Démares et al., 2016; Mengoni Goñalons and Farina, 2018). This could suggest a change in sensory perception, such as a lowering of sucrose responsiveness or odour perception. For example, neonicotinoids can impair sensory perception of odour in honeybees, which can be captured as a learning impairment (Andrione et al., 2016; Mustard et al., 2020), and sucrose responsiveness has been shown to be affected by neonicotinoids (Demares et al., 2018) and tau-fluvalinate (Frost et al., 2013), among others. Alternatively, glyphosate may not directly affect a bumblebee’s ability to detect sucrose or scent, but may indirectly affect their motivation and desire to feed, as also found for other pesticides such as imidacloprid (Cresswell et al., 2012, 2014) and clothianidin (Thompson et al., 2015). Some research also suggests that glyphosate can have an effect on the honeybee gut microbiome (Motta et al., 2018; Cullen et al., 2023). Therefore, bumblebees exposed to glyphosate may be more likely to become full or have digestion issues resulting in a reduced appetite and therefore reduced motivation to respond. Additionally, Muth and Leonard (2019) found that after imidacloprid exposure, there was no bumblebee learning impairment but there was a reduction in feeding and foraging ability potentially resulting from impairments to motivation and sensory systems. A similar result was found by Lämsä et al. (2018) where imidacloprid was found to reduce the motivation of bumblebees to forage. Whatever the mechanism behind the reduced responsiveness following glyphosate exposure observed here, it could have knock-on effects on foraging behaviour. It is therefore important to understand the implications of the reduced responsiveness resulting from exposure to glyphosate with regard to wider foraging behaviour, and how this links to colony fitness.

Interestingly, the reduction in responsiveness in glyphosate-treated bees was not observed in the commercial formulation Roundup Biactive treatment, which contained the same concentration of glyphosate. Similarly, impacts of prothioconazole AI on learning level were not seen in the Proline formulation treatment. This suggests that when herbicides and fungicides are in formulation there may be interactions between the active ingredient and the co-formulants which modify the action of the active ingredient, in this case reducing its impact. Some research indicates that when comparing active ingredients and commercial formulations, there can be differences in responses as a result of the co-formulants (Williams et al., 2020; Straw and Brown, 2021). Our findings echo this previous work, and are of particular interest as this research area is small (Straw et al., 2022). As we have very little knowledge as to what ingredients such formulations contain, it is difficult to draw any conclusions as to what this could mean in general for commercial formulations and bees, but it demonstrates that the differing impacts of active ingredients and formulations is a key area for further research.

The reduction in responsiveness as a result of exposure to the glyphosate AI meant that the sample size in the glyphosate treatment of bees that successfully learnt the association was too small to test any potential impacts on learning. However, other research does suggest that glyphosate can affect learning behaviour in honeybees. For example, Balbuena et al. (2015) found that glyphosate can impair navigational ability, suggesting it may affect the navigational components of the honeybee brain in some way, while Mengoni Goñalons and Farina (2018) found that 9 day old honeybees had impaired olfactory learning resulting from exposure to glyphosate, but older honeybees did not. As bumblebees and honeybees can differ in their sensitivity to pesticide exposure (Cresswell et al., 2012; Woodcock et al., 2017), it would be important to fully assess the impacts of glyphosate on bumblebee learning in the future.

Although our knowledge of fungicides and the potential implications of exposure to fungicides for bees is increasing, the fungicide prothioconazole is greatly understudied (Cullen et al., 2019) and as such needs more research attention. This is particularly important as it is widely used (López-Ballesteros, et al., 2022) and residues can be found in nectar and pollen (Wallner, 2009; Roszko et al., 2016; Bokšová et al., 2021), thereby resulting in oral exposure of bees. We found there was consistently no impairment in learning ability caused by prothioconazole across experiments two and three, which provides strong evidence that prothioconazole may not cause reductions in olfactory learning. However, we did observe in experiment two that prothioconazole­-treated bees that had learnt the association displayed it more often (e.g. had a higher learning level) compared with control bees. This could demonstrate that despite there being no impairment, prothioconazole could change the behaviour of bees. Stressors when present in a low level can result in a hermetic response, meaning that there is an overcompensation whilst the affected system is repairing, which could be what is observed here (Cutler et al., 2022). However, it did appear these patterns could have been influenced by just one bee, and as such further research would be needed to determine this with certainty, particularly given the small sample size. Other research has suggested that hormetic effects have been produced by related fungicides in the sterol biosynthesis inhibitor family, causing increased survival in exposed bumblebees (Tosi and Nieh, 2019), while other pesticides such as coumaphos and imidacloprid have also been shown to enhance learning (Williamson et al., 2013), and thiamethoxam can increase bumblebee flower visitation rates (Stanley et al., 2015b; Stanley and Raine, 2016). Similar hormetic effects have been reported for non-pesticidal compounds, such as caffeine (Wright et al., 2013). However, while this increase in learning level following prothioconazole exposure was found in experiment two, it was not observed for the same treatments in experiment three. This could be related to differences in experimental design, e.g. colony age and origin, or the fact that one experiment used full colonies and the other microcolonies. Either way, this could suggest that external factors may modify the impact of prothioconazole, or that there was in fact no true effect on learning, which would be interesting to investigate further.

Changes in learning are commonly associated with impairment of the parts of the bee brain responsible for these functions. For example, neonicotinoid insecticides target the nicotinic acetylcholine receptors and therefore can affect the entire nervous system; as a result, they can impair the mushroom bodies, which are associated with learning and memory (Zars, 2000). It is therefore unsurprising that exposure to neonicotinoids is associated with cognitive impairment in honeybees (Palmer et al., 2013), and many studies on neonicotinoids have found impaired learning ability in both honeybees and bumblebees (Williamson and Wright, 2013; Stanley et al., 2015a; Wright et al., 2015; Piiroinen and Goulson, 2016; Siviter et al., 2018). In comparison, little is known about the mechanisms of prothioconazole in bees. It has been found to cause physiological changes, such as impairment of cytochrome P450 pathways (Haas and Nauen, 2021; Haas et al., 2021) associated with detoxification, and as such it is possible it could also cause other physiological changes associated with cognition in bees, but more research is needed to determine what these are. Additionally, Haas et al. (2021) and Haas and Nauen (2021) demonstrated that there are differences in the extent to which azole family fungicides can affect cytochrome P450 pathways, showing that bees may respond differently to exposure to fungicides even within classes with the same mode of action. Together, these findings demonstrate that we need to better understand the mechanisms behind potential effects of fungicides on bees, given their diversity in modes of action.

Interestingly, we found body size to be an important predictor of responsiveness and learning in some cases. Other research does suggest that responsiveness can be linked with body size, as larger bumblebees have an increased capacity to come into contact with odour molecules and as such can be more sensitive to odours (Spaethe et al., 2007). This could explain why we observed that smaller bees were more likely to show non-responses in experiment one, although this was not observed in experiments two and three, and in fact the opposite was found in experiment two. This is in contrast to other findings and to the results of experiment three which suggest that bigger bees can be better at learning (Ings et al., 2005; Worden et al., 2005; Stanley et al., 2015a) and memory (Riveros and Gronenberg, 2009), although others also found no correlation with body size and learning, as we did with the majority of our results (Ings et al., 2005; Raine et al., 2006; Raine and Chittka, 2008). Ultimately, there was no difference in body size across any treatment in any of the experiments, so whilst body size did influence some aspects of learning, it did not have any effect on response to treatment.

Some research suggests that increased foraging experience can result in larger mushroom bodies, which are important for bumblebee learning (Riveros and Gronenbery, 2010). However, other research suggests that foragers do not always perform better than in-hive workers (e.g. Martin et al., 2018). Therefore, as the bees from these experiments had little to no foraging experience and for one of these experiments no bees left their colony, foraging experience would have been unlikely to have much of an effect. In addition, as selection of foragers or workers was consistent within experiments, any influence of foraging experience would have been the same across treatments.

For the formulations Roundup Biactive and Proline, we used both a treatment based on plant residues to represent the lower end of exposure and one based on spray rates to represent the higher end of field-relevant exposure. Interestingly, we found that colonies consumed significantly less sucrose solution when it was contaminated with the Roundup high treatment (L.J.T., M. Dacke, D.A.S. and L. Herbertsson, unpublished), but not Proline, meaning that bees may have reduced their own exposure to the glyphosate formulation ingredients. As such, we cannot state that the absence of an observed effect was directly related to the Roundup high treatment, as bees may have been able to mostly feed on their nectar stores and minimise consumption of the contaminated sucrose solution. This is also interesting with regards to the risk of exposure to this herbicide, in that bumblebees may avoid these higher concentrations on plants, and again this warrants further research.

In general, our study could be considered conservative with regards to the dose and route of exposure of the active ingredients and formulations used. Bees may be exposed to glyphosate through nectar and pollen simultaneously when foraging (Thompson et al., 2014), and glyphosate concentrations can be detected for at least 3–7 days after plant treatment (Thompson et al., 2014; 2022), which is a longer time period than tested here. Therefore, our exposure to a low concentration through nectar alone for 3–4 days could result in a lower exposure than might be expected in the field, and it would be interesting to test a range of glyphosate concentrations and chronic exposure periods within the field-relevant range. For prothioconazole, we have little knowledge of the residues bees may come into contact with (Zioga et al., 2020; Rondeau and Raine, 2022) in comparison to glyphosate. However, it has previously been found in nectar and pollen (Wallner, 2009; Roszko et al., 2016; Bokšová et al., 2021) and as such our exposure through nectar alone may be also be deemed conservative.

Whilst our findings provide an initial insight into how fungicides and herbicides may affect learning in bumblebees, we also have more to learn. Although the use of the PER is a well-accepted technique, this method only tested olfactory learning ability. In reality, bees interact with flowers which have a variety of signals, and PER alone does not allow for a full evaluation of how all sensory systems in the bee may be affected (Muth et al., 2019), and other learning types (e.g. visual) may be differentially impacted, which deserves further work. This is especially important to recognise as Helander et al. (2023) showed that fine visual discrimination but not olfaction conditioning in bumblebees was affected by glyphosate. Additionally, as we only trained bees to one scent, it is not possible to determine whether they learnt the association between the lavender odour and reward, i.e. scent and reward, or the association between air blowing over their antenna and the reward, and this could also be investigated further. Finally, as the exact mechanisms behind the impacts of herbicides on responsiveness remain unclear, it is uncertain whether there is a true effect on motivation or a change in sensory perception of the odour (e.g. Andrione et al., 2016) and this also warrants further work.

### Conclusions

From these experiments we can conclude that the commercial formulations Proline and Roundup Biactive may not pose a hazard to bumblebees with regards to olfactory learning, when receiving field-realistic short but chronic doses. However, we saw some effects of active ingredients not seen in their respective commercial formulations, which suggests that in some cases co-formulants could modulate the impacts of active ingredients on bees. Glyphosate has the potential to reduce responsiveness in bumblebees as a result of either sensory or motivational changes, whilst prothioconazole enhanced the learning level of bumblebees, although more research is required to understand this and elucidate the mechanisms behind the effects seen. How these changes relate to colony fitness and foraging is yet to be determined, and future research should focus on understanding these potential implications to better inform pesticide risk assessment, use and bee conservation.

## Supplementary Material

10.1242/jexbio.245180_sup1 Supplementary information
