# Supplementary material for: Contrasting effects of fungicide and herbicide active ingredients and their formulations on bumblebee learning and behaviour
Source: J Exp Biol. 2023 Mar 14;226(6):jeb245180. doi: 10.1242/jeb.245180 (PMC10112909; doi:10.1242/jeb.245180)
Supplement: Supplementary information [file jexbio-226-245180-s1.pdf]

Supplementary Materials and Methods

Memory assays: Harnessed bumblebees were left in a dark room for 2 hours after PER training. All bees were presented the odour after two hours to test their ability to remember the association. Memory retention was poor for all three experiments and sample size was not sufficient to perform analysis on how these pesticides effect memory. Three bees from Experiment one remembered the association, one from Roundup low, one from Roundup high and one from glyphosate AI. Four bees from Experiment two remembered the association, three from prothioconazole and one from control. And four bees remembered the association in Experiment three, two from prothioconazole AI and two from Proline low.

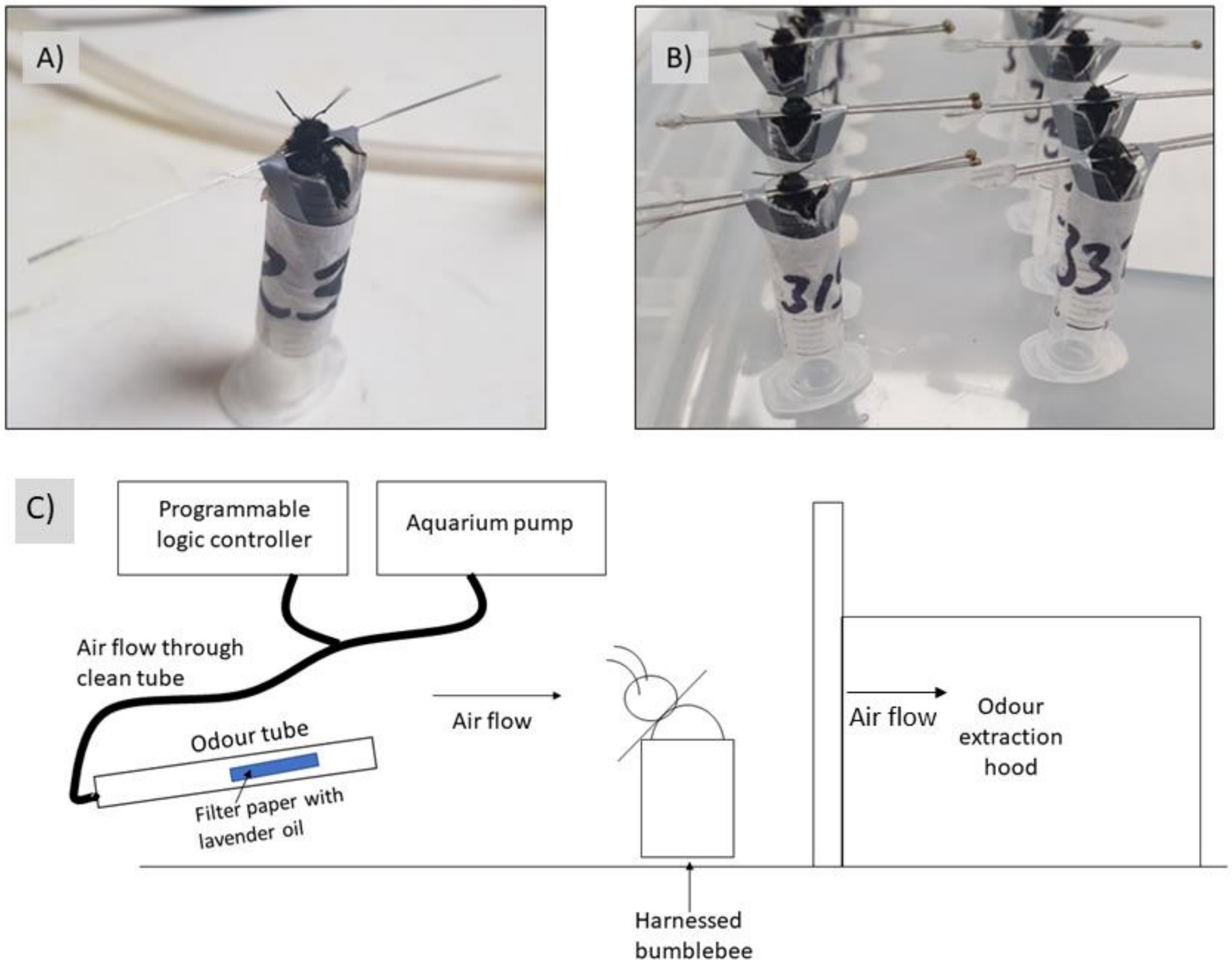

Fig. S1. Photos showing the difference between harnessing techniques A) in Experiment one and two and B) in Experiment three. We found that the anesthetization and harnessing methods of experiments one and two (photo A) were most effective. C) shows a diagram of the PER set up.

Table S1. showing full model puts and 95 % confidence intervals for all statistical analysis performed for Experiment One, Experiment Two and Experiment Three. Significant differences are highlighted in bold.

Experiment One: Herbicide Colony Level			Final model output					95 % confidence intervals		
Parameter	**Full model**	**Final model**	**parameter**	**Estimate**	**Standard error**	**Z value**	**P value**	**Lower CI**	**Upper CI**	**Model/ package**
Trainability	Learnt (1/0)~ treatment + intertegular distance + random: run/colony	Learnt (1/0)~ intertegular distance + random: colony/run	Intercept	-0.83	0.45	-1.82	0.07	-1.72	0.06	glmmTMB/
			Treatment: Glyphosate AI	-0.88	0.89	-0.98	0.33	-2.63	0.87	glmmTMB
			Treatment: Roundup high	0.67	0.72	0.94	0.35	-0.73	2.08	
			Treatment: Roundup low	0.42	0.64	0.66	0.51	-0.84	1.68	
Learning rate	Trial first learn association ~ treatment + intertegular	Trial first learn association ~ intertegular distance + random:	Intercept	-3.27	1.84	-1.78	0.08	-6.87	0.34	glmmTMB/ glmmTMB
			Treatment: Roundup high	0.01	0.21	0.03	0.98	-0.41	0.42	

Journal of Experimental Biology • Supplementary information

	distance + random: run/colony	run/colony	Treatment: Roundup low	0.41	0.21	1.9	0.06	-0.01	0.83	
			Intertegular distance	1.3	0.45	2.87	**0.004**	0.41	2.19	
Learning level	Number of learnt responses ~ treatment + intertegular distance + random: run/colony	Number of learnt responses ~ intertegular distance + random: run/colony	Intercept	8.59	3.57	2.41	0.16	1.59	15.58	glmmTMB/ glmmTMB
			Treatment: Roundup high	-0.19	0.42	-0.46	0.65	-1.01	0.63	
			Treatment: Roundup low	-0.64	0.43	-1.47	0.14	-1.49	0.21	
			Intertegular distance	-1.83	0.89	-2.06	0.04	-3.6	0.21	
Number of non-responses	Total number of non-responses ~ treatment + intertegular	Number of non-responses ~ treatment + intertegular distance +	Intercept	6.51	1.66	3.93	0	3.26	9.75	glmmTMB/ glmmTMB
			Treatment: glyphosate	1.14	0.38	2.95	**0.003**	0.38	1.89	

	distance + random: run/colony	random: run/colony	Treatment: Roundup low	0.34	0.39	0.86	0.39	-0.43	-0.76	
			Treatment: Roundup high	0.72	0.39	1.85	0.06	-0.05	1.48	
			Intertegular distance	-1.58	0.42	-3.8	**0.0001**	-2.39	-0.76	
Number of bees with a non response	Number of bees with a non-response ~ treatment + intertegular distance + (random: run/colony)	Number of bees with a non-response ~ treatment + intertegular distance + (random: run/colony)	Intercept	11.42	3.92	2.91	0.004	3.74	19.1	glmmTMB/ glmmTMB
			Treatment: glyphosate	2.12	0.8	2.65	**0.01**	0.55	3.69	
			Treatment: Roundup low	0.69	0.7	0.99	0.32	-0.68	2.05	
			Treatment: Roundup high	1.25	0.8	1.56	0.12	-0.32	-1.18	
			Intertegular distance	-3.18	1.02	-3.12	**0.001**	-5.18	-1.18	

Journal of Experimental Biology • Supplementary information

Intertegular distance	Intertegular distance ~ treatment + (random: run/colony)	Intertegular distance ~ treatment + (random: run/colony)	Intercept	3.85	0.06	61.68	00.00	3.73	3.98	lme/ nlme
			Treatment: glyphosate	0.18	0.1	1.73	0.09	-0.02	0.38	
			Treatment: Roundup low	-0.06	0.09	-0.68	0.5	-0.14	0.29	
			Treatment: Roundup high	0.07	0.1	0.71	0.49	-0.24	0.12	
Experiment Two (Prothioconazole Colony Level)			Final model output					95 % confidence intervals		
Parameter	**Full model**	**Final model**	**parameter**	**Estimate**	**Standard error**	**Z value**	**P value**	**Lower CI**	**Upper CI**	**Model/ package**
Trainability	Learnt (1/0)~ treatment + intertegular distance + random: run/colony	Learnt (1/0)~ treatment + random: colony/run	Intercept	-0.69	0.39	-1.79	0.07	-1.45	0.07	glmmTMB/

Journal of Experimental Biology • Supplementary information

			Treatment: prothioconazole	0.51	0.52	0.98	0.33	-0.51	1.53	glmmTMB
Learning rate	Trial first learn association ~ treatment + intertegular distance + random: run/colony	Trial first learn association ~ intertegular distance + random: run/colony	Intercept	2.29	0.1	22.81	<2e-16	2.1	2.49	glmmTMB/ glmmTMB
			Treatment: Prothioconazole	0.03	0.13	0.24	0.81	-0.23	0.29	
Learning level (including outlier)	Number of learnt responses ~ treatment + intertegular distance +	Number of learnt responses ~ treatment +intertegular distance	Intercept	5.43	2.26	2.4	0.02	1	9.86	glmmTMB/ glmmTMB

Journal of Experimental Biology • Supplementary information

	random: run/colony	random: run/colony	Treatment: prothioconazole	0.69	0.28	2.45	**0.01**	0.14	1.24	
			Intertegular distance	-1.28	0.61	-2.09	**0.04**	-2.48	-0.08	
Learning level (removing outlier)	Number of learnt responses ~ treatment + intertegular distance + random: run/colony	Number of learnt responses ~ treatment + random: run/colony	Intercept	0.69	0.24	2.95	0.003	-0.55	8.94	glmmTMB/ glmmTMB
			Treatment: Prothioconazole	0.38	0.31	1.25	0.21	-0.1	1.12	
Number of non-responses	Total number of non-responses ~ treatment + intertegular distance +	Number of non-responses ~ intertegular distance + random: run/colony	Intercept	-17.66	5.73	-3.08	0.002	-28.88	-6.44	glmmTMB/ glmmTMB
			Treatment: Prothioconazole	20.27	7.71	2.63	**0.008**	5.17	35.37	

Journal of Experimental Biology • Supplementary information

	random: run/colony		Intertegular distance	4.55	1.45	3.14	**0.002**	1.71	7.4	
			Treatment*Intertegular distance	-5.3	1.99	-2.67	**0.0018**	-9.2	-1.41	
Number of bees with a non response	Number of bees with a non-response ~ treatment + intertegular distance + (random: run/colony)	Number of bees with a non-response ~ intertegular distance + (random: run/colony)	Intercept	-29.98	12.6	-2.38	0.02	- 54.67	-5.29	glmmTMB/ glmmTMB
			Treatment: Prothioconazole	33.65	14.07	2.39	**0.02**	6.07	61.23	
			Intertegular distance	7.49	3.24	2.31	**0.02**	1.14	13.85	
			Treatment* Intergular distance	-8.8	3.66	-2.41	**0.02**	- 15.97	-1.64	

Journal of Experimental Biology • Supplementary information

Intertegular distance	Intertegular distance ~ treatment + (random: run/colony)	Intertegular distance ~ treatment + (random: run/colony)	Intercept	3.72	0.05	77.22	0	3.63	3.82	lme/ nlme
			Treatment: Prothioconazole	0.074	0.07	1.12	0.27	3.63	3.82	lme/nlme
Experiment Three: Fungicide Microcolony Level			**Final model output**					**95 % confidence intervals**		
Parameter	Full model	Final model	parameter	Estimate	Standard error	Z value	P value	Lower CI	Upper CI	Model/ package
Trainability	Learnt (1/0)~	Learnt (1/0)~	Intercept	0.41	0.55	0.75	0.46	-0.66	1.48	glmmTMB/

Journal of Experimental Biology • Supplementary information

	treatment + intertegular distance + random: natal colony/ run/ microcolony	treatment + random: natal colony/ run/ microcolony	Treatment: Prothioconazole AI	-0.02	0.65	-0.04	0.97	-1.29	1.24	glmmTMB
			Treatment: Proline low	-0.37	0.68	-0.54	0.59	-1.69	0.96	
			Treatment Proline high	-0.84	0.73	-1.16	0.25	-2.27	0.58	
			Treatment: Sucrose	0.28	0.71	0.4	0.69	-1.11	1.68	
Learning level	Number of learnt responses~ treatment + intertegular distance + random: natal colony/	Number of learnt responses ~ treatment+ intertegular distance + random: natal colony/ run/	Intercept	-0.33	0.89	-0.37	0.71	-2.08	1.42	glmmTMB/ glmmTMB
			Treatment: acetone	-0.41	0.28	-1.43	0.15	-0.96	0.15	
			Treatment: prothioconazole	-0.51	0.28	-1.8	0.07	-1.07	0.05	

Journal of Experimental Biology • Supplementary information

	run/ microcolony	microcolony	Treatment: Proline low	-0.59	0.3	-1.98	0.05	-1.17	-0.01	
			Treatment: Proline high	-0.43	0.34	-1.25	0.21	-1.09	0.24	
			Intertegular distance	0.51	0.23	2.18	**0.03**	0.05	0.97	
Learning rate	Trial first learn association ~ treatment + intertegular distance + random: natal colony/ run/ microcolony	Trial first learn association ~ treatment + random: natal colony/ run/ microcolony	Intercept	2.03	0.14	14.7	<2e-16	1.76	2.3	glmmTMB/ glmmTMB
			Treatment: acetone	0.09	0.17	0.51	0.61	-0.25	0.42	
			Treatment: Prothioconazole	0.09	0.16	0.54	0.59	-0.23	0.41	
			Treatment: Proline low	0.05	0.18	0.25	0.81	-0.32	0.41	
			Treatment: Proline high	-0.08	0.21	-0.39	0.7	-0.32	0.41	

Journal of Experimental Biology • Supplementary information

Number of non-responses	Total number of non-responses ~ treatment + intertegular distance + random: run/colony	Total number of non-responses ~ intertegular distance + random: run/colony	Intercept	1.17	0.33	3.55	0	0.53	1.82	glmmTMB/ glmmTMB
			Treatment: acetone	0.09	0.48	0.21	0.83	-0.78	0.97	
			Treatment: Prothioconazole	0.47	0.41	1.13	0.26	-0.35	1.28	
			Treatment: Proline low	0.05	0.46	0.1	0.92	-0.85	0.94	
			Treatment: Proline high	0.7	0.43	1.61	0.1	-0.15	1.55	
Number of bees with a non response	Number of bees with a non-response ~ treatment + intertegular distance + (random: run/colony)	Number of bees with a non-response ~ intertegular distance + (random: run/colony)	Intercept	0.29	0.44	0.65	0.51	-0.58	1.15	glmmTMB/ glmmTMB
			Treatment: acetone	-0.05	0.6	-0.08	0.94	-1.22	1.12	
			Treatment: prothioconazole	0.49	0.57	0.86	0.39	-0.63	1.61	

Journal of Experimental Biology • Supplementary information

			Treatment: proline low	0.34	0.62	0.55	0.58	-0.88	1.56	
			Treatment: proline high	0.97	0.64	1.51	0.13	-0.29	2.22	
Intertegular distance	Intertegular distance ~ treatment + (random: run/colony)	Intertegular distance ~ treatment + (random: run/colony)	Intercept	3.74	0.11	35.56	0	3.53	3.94	lme/ nlme
			Treatment: Prothioconazole	-0.1	0.09	-1.18	0.24	3.54	3.92	

Journal of Experimental Biology • Supplementary information

			Treatment: Proline low	0.01	0.097	0.13	0.9	-0.28	0.07	
			Treatment: Proline high	-0.031	0.11	-0.3	0.77	-0.17	0.2	
			Treatment: Sucrose	0.007	0.09	0.074	0.94	-0.18	0.2	

Journal of Experimental Biology • Supplementary information

Table S2. Full Tukey output from Experiment One – Herbicide Colony Level. Significant differences are highlighted in bold.

Parameter	contrast	Estimate	t-ratio	p value
Number of non-responses (Experiment One)	Control – glyphosate AI	-1.14	-1.14	**0.02**
	Control – Roundup low	-0.72	-0.72	0.26
	Control – Roundup high	-0.34	-0.34	0.83
	Glyphosate AI – Roundup low	0.42	0.42	0.67
	Glyphosate AI – Roundup high	0.80	0.80	0.12
	Roundup low – Roundup high	0.38	0.38	0.73
Number of bees which had a non-response (Experiment One)	Control – glyphosate AI	-2.12	-2.65	**0.05**
	Control – Roundup low	-1.25	-0.99	0.76
	Control – Roundup high	-0.69	-1.56	0.41
	Glyphosate AI – Roundup low	0.87	1.80	0.28
	Glyphosate AI – Roundup high	1.43	1.02	0.74
	Roundup low – Roundup high	0.56	-0.72	0.88
Learning level (Experiment Two – including outlier)	Control - Prothioconazole	-0.69	-2.45	0.02

Table S3. The concentrations of active ingredient converted into molar.

Treatment	Concentration (mg kg^{-1})	Concentration (µMolar)
Glyphosate	1	5.9
Prothioconazole	0.3	0.87

Journal of Experimental Biology • Supplementary information
